# A Pilot Study in Cameroon to Understand Safe Uses of Pesticides in Agriculture, Risk Factors for Farmers’ Exposure and Management of Accidental Cases

**DOI:** 10.3390/toxics5040030

**Published:** 2017-11-01

**Authors:** Guy Bertrand Pouokam, William Lemnyuy Album, Alice S. Ndikontar, Mohamed El Hady SIDATT

**Affiliations:** 1 Laboratory of Food Safety, Biotechnology Center, University of Yaounde 1, Cameroon; 2 National Focal Point Rotterdam Convention, Ministry of Environment, Nature Protection and Sustainable Development, Yaounde, Cameroon; lemnyuy@yahoo.com; 3 National Technical Coordinator of Project. Disposal of POPs, Obsolete Pesticides and Strengthening Sound Pesticide Management in Cameroon, Food and Agriculture Representation in Cameroon, Yaounde, Cameroon; Alice.NdikontarSiben@fao.org; 4 Rotterdam Convention Secretariat, Food and Agriculture Organization Regional Office for North-Africa, Tunis, Tunisia; MohamedelHady.Sidatt@fao.org

**Keywords:** pesticides, approval, intoxication, poisoning, surveillance

## Abstract

Chemical pesticides are widely used in Cameroon for agricultural production. In 2015, more than 600 pesticide products were approved for use in various foodstuffs. Much misuse of these chemicals by farmers has been documented in rural and urban settings. This pilot study aims to contribute to the improvement of the health of the population and the environmental preservation by identifying pesticide-poisoning cases, the most incriminated products and critical risk factors of exposure. Questionnaires were administered to pesticide vendors, farmers and health personnel, and observations made on farmers’ practices at their work places. From July to September 2016, 24 villages from five sites, representing the most important agricultural production areas of the five agro-ecological zones of Cameroon, were visited. In total, 519 people were interviewed: 412 farmers, 69 pesticide vendors and 38 health personnel. A total of 180 pesticide formulations out of 610 registered in 2015 were said to be used by farmers. In the 38 health centers visited, 56 cases of pesticide poisonings and intoxications were reported between 2011 and 2016. Paraquat-, glyphosate-, cypermethrin- and metalaxyl-formulated pesticides were the most incriminated. In total, 78% of poisoning cases were accidental, 12% suicide attempts, 4% criminal. Entry of pesticide products from neighboring countries needs to be better regulated, and the quality of pesticides sold on the market should be monitored periodically. Empty pesticide containers should be recuperated from smallholder farmers. Authorities should set up a harmonized pesticide-poisoning management procedure, and create a toxico-vigilance system for surveillance cases and preventive actions.

## 1. Introduction

The FAO (Food and Agriculture Organization of the United Nations) defines pesticide as any substance or mixture of substances of chemical or biological ingredients intended for repelling, destroying or controlling any pest, or regulating plant growth [1]. Active pesticide molecules belong to various chemical groups and are able to interact with the normal functioning of living systems, and therefore can alter various metabolic pathways to create numerous pathologies. Pesticides can be classified based on the pest they control (fungicides, insecticides, herbicides), or on the chemical class (organochlorines, pyrethroids, organophosphates) [2].

The database of pesticides approved for agricultural uses in Cameroon in 2015 contains more than 600 pesticide products [3], with dominant products being insecticides (33.93%); herbicides (26.55%); fungicides (24.26%); insecticides–fungicides (4.26%); nematicides (2.3%); growth regulators (1.48%); molluscides (1.15%); and insecticides–nematicides (1.31%). Types of formulation according to the international classification code revealed that up to 65% of approved pesticides belong to only four types of formulation: 28% EC (emulsifiable concentrate), 15.25% WP (wettable powder), 11.96% SC (suspension concentrate) and 11.96% SL (soluble liquid concentrate). These formulations are mixed with water then applied as sprays. Their toxicology classes, according to the World Health Organization classification, indicated that 3% of approved pesticides are in class Ib (highly hazardous), among which are insecticides and nematicides used to treat cotton, tomatoes, plantains, vegetables; 2% class Ia (extremely hazardous) for use in food storage; 32% class II (moderately hazardous); and 63% class III (slightly hazardous).

Pesticide poisoning is gradually becoming a major public health concern in Cameroon. Unfortunately, this problem remains under-addressed, mainly because of the poor understanding of its implications. Beyond the formal approval procedure for uses, no post-registration surveillance mechanism or toxicovigilance system exists to monitor accidents and intoxication that may occur [4].

However, much misuse and many risk factors that can result in serious health risks for farmers and the general population have been documented: presence of pesticide residues in most consumed foodstuffs [5], non-use of personal protective equipment and drinking-water contamination [6,7,8,9,10].

Pesticide poisoning can occur shortly or a long time after exposition. Poisoning can occur at home, on the farm, via an attempt of suicide, accidental ingestion, poisoning, alcohol contamination, or during spraying [11,12]. Farmers’ behaviors have been explored in order to better understand how their working environment, including exposure to pesticides, affects their health [13].

A pilot survey that was carried out in the ten regions of Cameroon between 2001 and 2002 demonstrated that low quality of spraying equipment was a non-negligible cause of accidents. Moreover, the absence of personal protective equipment plays a key role in the extent of body exposure. With regards to environmental health risk factors, it was found that more than 60% of smallholder farmers throw empty containers of pesticides in the nearby river, 25% wash their knapsack sprayers at the closest water point, and some even discharge the remaining pesticide preparation in the river [6]. The same authors indicated that the most used pesticides in the country were Paraquat and Glyphosate (herbicides), Cypermethrine and Chlorpyryfos (insecticides), and Metalaxyl and Maneb copper (fungicides). To keep pesticides out of reach of children, farmers usually bury pesticide bottles containing the remaining product on the farm until later use [10].

Similar studies with farmers in the Galim (west region) showed that only 2.1% of farmers wear personal protective equipment; more than 76% did not follow the user’s instructions written on the product label [14]. Similarly, Tinyami et al. (2014) [9] reported that, of tomato farmers in Buea (south-west region), 47.6% of farmers use pyrethrinoids and organophosphorus insecticides, 83.8% make use of knapsack sprayers to treat their plants, 76% did not use personal protective equipment and 85% were said to have experienced at least one symptom of acute pesticide toxicity after pesticide handling.

Concerning the environmental impact of the massive use of pesticides by fruit and vegetable farmers in urban and peri-urban areas of Bamenda (north-west region), environmental pollution has been found to be a huge concern because of the non-adherence to the regulations on the usage of pesticide products. In addition, farmers reported many cases of acute pesticide poisoning: itches, skin burn, eye problems, cough, chest pain, nausea, vomiting, headache and dizziness. To attenuate the harmful effects of pesticides on their bodies, farmers claim to have developed numerous strategies depending on the situation: they drink a charcoal solution, remove their dresses, wash their hands or their bodies, take red palm oil, drink honey or drink some beers [8].

These uses and misuses of agricultural pesticides can have a significant impact on environmental pollution, and finally end up in foodstuffs. A study on the misuse of pesticides in the north region of Cameroon by farmers [7] revealed that organochlorine residues (lindane, alpha-endosulfan and beta-endosulfan), organophosphorus residues (malathion, pirimiphomethyl), synthetic pyrethrinoids (permethrine) and carbamates (carbofuran) are found in maize and millet in certain localities of that region at levels higher than the recommended Maximum Residue Limits (MRLs). Nine pesticide residues were detected in cooked foods: atrazine (spices), chlorothalonil (vegetables), cypermethrin (tomatoes), deltamethrin (bread), endosulfan (tomatoes, vegetables), malathion (wheat doughnut), pirimiphos-methyl (spaghetti), dithiocarbamates (papaya, pineapple, spices) and chlordecone (tomatoes) [5].

The above described situation suggests poor understanding of pesticide health risks by farmers, significant environmental pollution and human exposure on farms, a non-negligible prevalence of pesticide poisoning cases, as well as chronic pesticide-related diseases.

To once again highlight this problem, and better protect the health of the population and the environment, national authorities within the framework of the Rotterdam Convention on the management of hazardous chemicals subjected to international trade initiated a pilot study in Cameroon. This study aimed at describing the distribution paths and handling practices of pesticides in the country, to survey cases of suspected pesticide poisoning of farmers and identify possible risk factors that could exacerbate human and environmental effects.

## 2. Materials and Methods

### 2.1. Areas of the Study

The country is divided into five different agro-ecological zones, from the southern to the northern part of the country: (i) the bi-modal humid forest zone (Equatorial Guinea), which is characterized by two rainy seasons per year. Its main products are cocoa, coffee, plantain, maize, and pineapples; (ii) the humid mono-modal forest zone (between Equatorial Guinea and tropical humid), with one important rainy season. Its main products are cocoa, coffee, banana, plantain, ginger, and sweet pepper. In the inner part of this zone, we observed the western highland with important rainy seasons and very fertile soil (cocoa, coffee, maize, beans, potatoes, fruits and vegetables); (iii) the high savanna zone (tropical humid) situated between the forest and savanna zones (maize, millet, cotton, yams, and potatoes); (iv) the Soudano–Sahelian zone characterized by very short rainy seasons (cotton, yams, onion, sesame, and groundnuts); and (v) the western highland, which is very similar to the humid forest mono-modal zones.

These five agro-ecological zones are characterized by different climatic conditions, which affect the types of agricultural products and agricultural practices in each zone, and thus the usage of pesticides. The selection of these five zones was also based on their agricultural contributions to the national food supply. In addition, each site surveyed (as indicated in Table 1) was included for specific food items to represent a wide range of pesticides in circulation. The survey was extended to the villages surrounding the main sites as farmers in these villages are important contributors to the overall supply of food in the town.

### 2.2. Type of Study

We carried out an exploratory epidemiological study combining questionnaires administered to three target populations, and observations of working practices on the farm for farmers and in the shop for the pesticide distributors.

### 2.3. Target Populations

Target populations were in three categories: pesticide users (farmers), pesticide distributors (salesmen and women) and health personnel in private and public health facilities in the surveyed area.

### 2.4. Sampling

**Figure 1:**
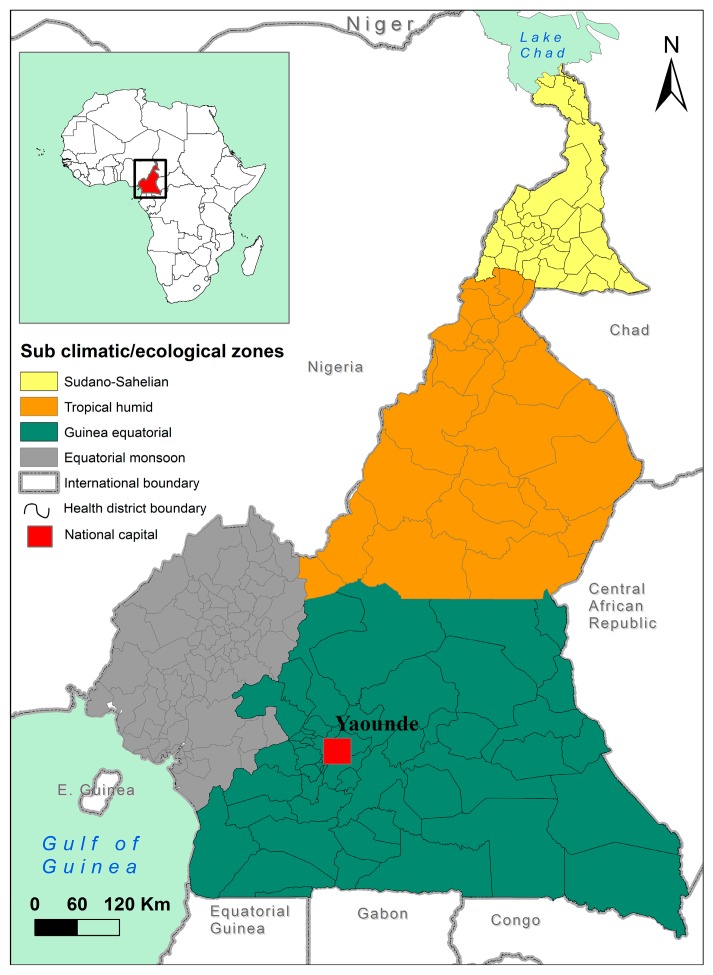
Map of Cameroon, showing the five agro-ecological zones.

In each agro-ecological zone (Figure 1), the main agricultural production and distribution sites were included as they represent the most important concentration of pesticide salesmen; then, in consultation with the divisional officer of the ministry in charge of agriculture, the cartography of villages and farmers’ groups in the area was verified in order to identify the major items produced and their origins. Therefore, some farmers’ groups were selected randomly based on their localization and the items they produced. Appointments were made to visit members of the farmers’ union. Moreover, visits were also made to individual farms of non-members of any union. Each group received its own questionnaire.

#### 2.4.1. Pesticide Distributors (Salesmen)

Salesmen present at the time of survey in selected sites and villages visited were approached for an interview. Interviewers were authorized by the ministry in charge of agriculture and were accompanied in the field by an officer of that ministry to facilitate not only the identification of sale points, but also their approval to respond to the questionnaire. In addition, pictures were taken of pesticide products available in the shop, and names of pesticides written down.

#### 2.4.2. Farmers (Pesticide Users)

Farmers were visited on their farms, at home, in their organization’s headquarters and at the marketplace. Questionnaires for pesticide users were administered and when on farms, observations were done on certain practices and risk factors of pesticide poisoning.

#### 2.4.3. Health Personnel

With the authorization letter of the Ministry of Public Health, the survey in each site started with a working session with the representative of that ministry in the zone. Then, the cartography and list of health centers were verified, and health facilities visited. Depending on the category of the healthcare center, some health personnel present during our visit, involved in the chain of treatment and available at that time, were interviewed. The aim of the questionnaires was to capture their perceptions and cases of pesticide poisoning received in their health structures.

### 2.5. Questionnaire Administration

Questionnaires were administered by trained interviewers. The questionnaire was developed by the Rotterdam Convention of the Food and Agriculture Organization during similar studies in other countries. During a national workshop in preparation of the field survey, these questionnaires were reviewed and adapted to reflect the realities and priorities of the country. The questionnaires were applied to 519 individuals from 24 villages investigated, in the five agro-ecological zones selected as indicated in Table 2.

## 3. Results and Discussion

### 3.1. Distributors

**Figure 2:**
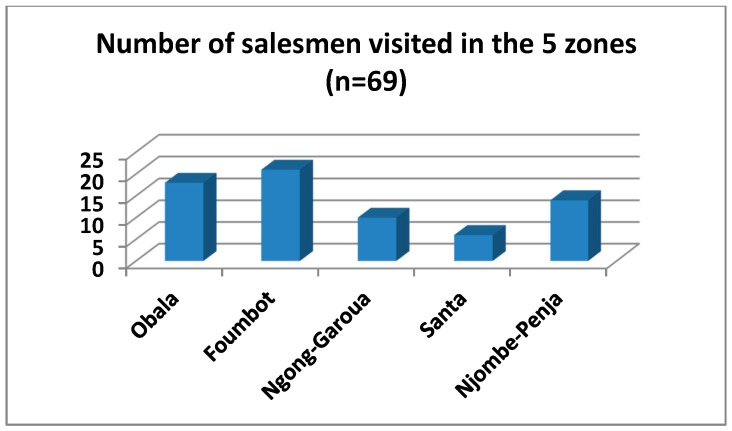
Number of pesticide sale-points visited.

In total, 69 pesticide sale-points were visited in the five agro-ecological zones (Figure 2). Of the 69 persons surveyed, 35 were shop assistants and 17 shop owners. The west region had the greatest concentration of pesticide distributors (21), as many importers have a representation there.

In the northern regions, approved pesticides are difficult to find because of the absence of importers and the proximity with Nigeria. In this part of the country, pesticides are sold during the market day. Therefore, salesmen travel to different villages according to the markets’ calendar in the area. National regulation prohibits the sale of pesticides as goods; unfortunately, it is the most common sales strategy in the northern part of the country.

Pesticide vendors indicated that 16% of products sold are returned by farmers, dissatisfaction with the product efficacy accounting for 15% of returns and defective wrapping paper for 1.4%.

In total, 55% of pesticide shops do not have any store or warehouse. In practice, they have limited quantities, and newly supplied products stay in a corner of the shop. So, they have no knowledge on good storage practices as stipulated by the regulations that regulate the sale and distribution of phytosanitary products in Cameroon. In the same vein, the regulations stipulate that sale-points should be dedicated only to phytosanitary products; unfortunately, more than 50% of sale-points visited were not specialized. They all had a mixture of hardware, cosmetic and phytosanitary products, or often, a typical shop with different kinds of food products, and a corner for phytosanitary products.

In total, 65% of salesmen did not have a registration form for carrying inventory and for product traceability, and 76% had no security information MSDSs (Material Safety Data Sheets) for the products they had. In the northern part of the country, more than half of the sale-points repack pesticides into a new bottle. In total, 17% were confirmed to repack pesticides in small quantities to meet farmers’ demands.

Overall, 94% of sellers were aware of the harmful effects of pesticides, but only 55% were able to cite some of these effects.

### 3.2. Farmers

**Figure 3:**
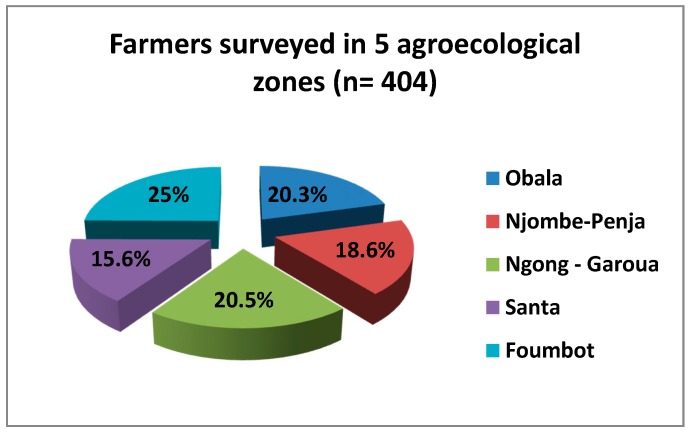
Distribution of farmers surveyed in the five agro-ecological zones.

A total of 404 farmers were surveyed in the five agro-ecological zones visited, as indicate in Figure 3.

Farmers were interviewed in their farms, homes, headquarters of their union and market places. Farmers’ ages varied between 20- and 79-years old. Table 3 gives the mean, minimum and maximum age of farmers surveyed.

In total, 69% of farmers visited were women. They are involved in all steps of the farm work. It was noticed that, in the northern part of the country, more than 94% of pesticide application is carried out by men. In total, 51% of farmers stopped their education at the primary-school level, and only 10% attended university. Moreover, 80% of persons interviewed indicated farming as their sole activity. In total, 62% of them use less than two hectares of farming land, with less than five workers, who most often are members of the same family.

#### 3.2.1. Main Cultures

Agricultural products vary between villages within the same zones and between agro-ecological zones; however, the five main crops represent between 62% and 81.5% of the overall produce, as seen in Table 4.

#### 3.2.2. Most Used Pesticides

The most widely-used pesticide is related to the main agricultural products in the main surveyed sites and surrounding villages. Table 5 is a synopsis of the most used pesticides (top 5). The cumulative percentage of these top-five pesticides represents from 40% to 80% of the overall pesticides in circulation in those zones. In Obala sites, where cocoa is the main culture, fungicides containing metalaxyl (Ridomil, Callomil and Plantomil) are the most used products, followed by insecticides (Actara, Onex, Parastar and Lamida Gold). Some of the active substances are metalaxyl and imidaclopride. It was also noted that the insecticide Lamida Gold is used in the Obala zone, while the product is homologated for use on tomatoes; this may suggest possible misuse. In the four other zones, Gramoxone and Roundup appear to be the most used pesticides. However, it was noted that Gramoxone was no longer approved for use in Cameroon. However, because Gramoxone has been used for a long time by certain farmers, when purchasing their pesticides, some farmers continued to request Gramoxone, without knowing that it is no longer in circulation. Salesmen say that in such cases, they provide farmers with a paraquat equivalent.

Glyphosate products are the most used herbicide in each zone (Roundup and Glyphader). It was difficult to identify certain products used by farmers because many did not remember the name of the pesticide, and they used local names such as “timides” to generally designate insecticides.

The specificity of phytosanitaries and pesticides used in the northern part of the country is quite obvious compared to other regions, but reflects the particularities of their production. Atrazine and Diuron are mostly used here, but they are not found elsewhere; both products are used for cotton production.

#### 3.2.3. Pesticide Risk Perceptions by Farmers

Concerning health risks related to pesticide exposure, 78% of farmers (*n* = 399) were said to be aware. In total, 15% of them consider that the most important risks are cutaneous problems, 13% by ingestion, 10% ocular difficulties. Cutaneous diseases are perceived as the most important problem in Obala, where cocoa is the most dominant crop and where atomizers are used for spraying; and also in Njombe–Penja, where aerial sprays are made by certain industries. In Foumbot, Santa and Ngong, inhalation and ingestion came first.

In total, 49% (*n* = 281) of farmers declared that pesticides are useful for a good production; 4.2% were aware that they can be hazardous for human health and 2% that they can be hazardous for the environment.

When a pesticide is opened and not fully used, 59% of farmers keep the rest at home for reuse, and 16% kept in the farm. In total, 46% of farmers (*n* = 389) dispose of empty packages of pesticides on their farms, 7.2% bury them on the farm, 27.5% burn them, 6.2% keep them at home and 3% reuse empty packages for domestic purposes.

The labelling of these pesticide products encourages users to return empty packages to the distributors, but unfortunately, no mechanism in place to help farmers, who are mostly in rural areas and own small farms in Cameroon. In total, 90% (*n* = 373) of farmers indicated that they have no incentive to return empty pesticide packages.

In total, 80% of farms are near to a water point (river, well, backwater), and 63% of these water points are less than 25 m from farms. In total, 41% of farmers used water from these water points for domestic uses. After pesticide treatment, the rest of the products are poured in the nearby water points and sprayers are also washed there. As possible effects of pesticide disposal on their farm, 56% had witnessed the disappearance of certain living organisms: birds, fish, lizards, spiders, snakes, caterpillars, locusts, squirrels, butterflies, flies, grasshoppers, wasps, ants, frogs, snails, bees, mice, rats, antelopes, partridges, monkeys and guinea-fowls.

#### 3.2.4. Suspected Poisoning Cases

In relation to the question of whether they have ever experienced a pesticide poisoning situation, 40.3% of farmers (*n* = 395) reported having had at least one case. Cutaneous (eye and skin) and inhalation problems appear to be most common, as shown in Table 6.

Once exposed to a pesticide, farmers based in rural areas declared using different types of first-aid measures, depending on the case. It was noted that they were not aware of the warning and measures written on the product label. Commonly used actions include the following: for cutaneous contact, wash the contaminated part with clean water or clean water and soap; for ingestion, swallow red oil or drink milk, local white wine or citrus juice; for eye contact, wash with water. After these first-aid actions, they may consider going to the hospital depending on the evolution of the case.

In total, 32.8% of farmers interviewed (*n* = 381) asserted to have witnessed a pesticide accident. In total, 85% of victims were male, with age ranging from 2 to 84 years old. In total, 54.3% of cases were cutaneous problems, against 26.7% ingestion and 17.2% inhalation.

#### 3.2.5. Most Incriminated Pesticides

**Figure 4:**
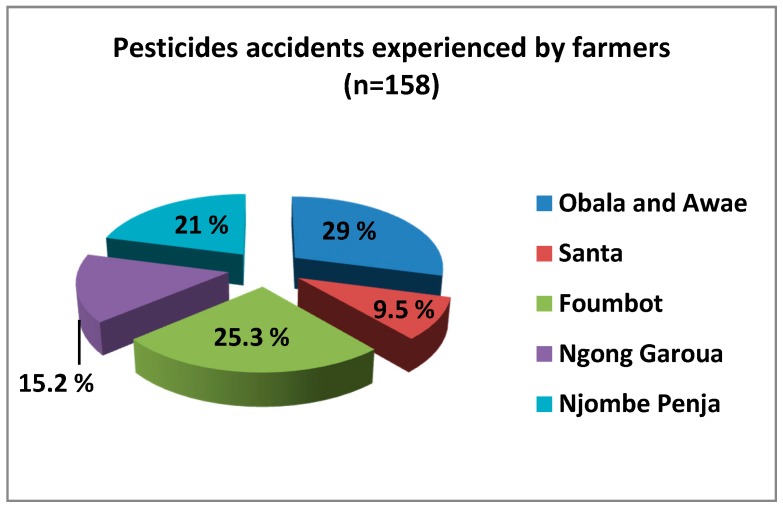
Cases of pesticide accidents in the five agro-ecological zones.

Concerning themselves, 158 farmers interviewed declared to have experienced at least one case of pesticide accident during manipulation, as shown in Figure 4.

Each of the agro-ecological zones was affected, and 80% of persons involved were male.

In Ngong–Garoua, 24 cases were declared by farmers, Roundup herbicide was cited six times (five cutaneous and one inhalation), Gramoxone in four cases (two inhalation, one cutaneous and one eye contact), three cases for Atrazine (two cutaneous and one inhalation) and four cases for Thioral (cutaneous). In seven other cases, farmers were not able to identify the product. Table 7 gives a synopsis of the most incriminated pesticides by farmers and by agro-ecological zones.

Among the most frequent signs and symptoms that appear after exposure to pesticides are itching and irritation, skin and eye problems, respiratory stress, tiredness, headache, vomiting and diarrhea.

Only 51.8% of farmers wear personal protective equipment, and moreover, even this equipment is incomplete or inappropriate. For example, 11.7% wore only boots. For plant treatment, 85% used a sprayer of 15 liters, 2% atomizers and 2% hand sprayer (2 liters). In total, 70% of farmers sprayed less than 10 liters per hectare, after dilution with water (89%). Pesticide treatments are done early in the morning, before 10 a.m. (for 87% of farmers). First treatments are applied between March and May for 60% of them, and last treatment between August and November for 58%.

Concerning the number of years they have been using pesticides, 33% of farmers said between 10 and 19 years, 32% between 1 and 9 years, 22% between 20 and 29 years, 20% between 30 and 39 years, 4% between 40 to 49 years and 1% for more than 50 years. Moreover, 57% affirmed not to be aware of good agricultural practices.

Despite their understanding of health risks from pesticide handling and previous cases of accidents experienced, 80% of farmers did not have any medical follow-up. Some of the farmers working with enterprises, specifically in the Njombe–Penja and Ngong–Garoua regions, were affiliated to a local health center where they were admitted for medical examination. In addition, few enterprises were found to have a health center for first-aid treatment and medical follow-up.

#### 3.2.6. Health Personnel

**Figure 5:**
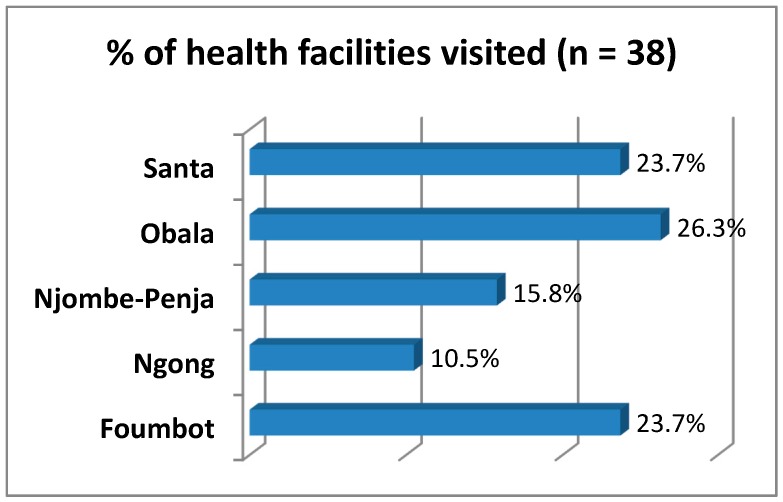
Distribution of health facilities.

To understand the level of preparedness of local healthcare centers and hospitals to manage pesticide poisoning cases, 38 health establishments in 24 villages of the five agro-ecological zones were visited (Figure 5).

In each locality where farmers were interviewed, nearby healthcare centers were also included and health personnel interviewed. In accordance with the organization of the Cameroonian health system, 38 healthcare establishments visited belonged to three categories: 40% were hospitals, 34% were classified health centers and 26% were integrated health centers.

Each time the survey team entered a healthcare establishment, an authorization letter signed by the Minister of Public Health was presented to the head of the structure. Then, a preliminary interview was done to understand the itinerary of patients and particularly patients complaining or showing signs and symptoms of pesticide poisoning. Thereafter, one to two health personnel suspected of being part of this chain were interviewed to gather information from their experience; where possible, registrations and archives for suspected cases were consulted. Overall, 42% of health personnel were medical doctors, and 21% nurses. The remaining personnel were nursing auxiliaries (8%), heads of the health centers (11%), pharmacists (3%) and midwives (2%).

In total, 61% of health personnel were not able to cite the name of at least one pesticide used by farmers of the locality. Others could cite, even using the common names, one to four of them. In total, 38 health workers from the 24 villages cited only 17 pesticide formulations out of the 600 approved for use in the country (Actara, Dursban, Diuron, Gramoxone, glyphosate, Roundup, Lamida, Manisan, Mocab cethomophos, Pyriforce, oxaplant, Ridomil, callomil, timide, Manozan, Kung-fu). In total, 4% cited names of fertilizers instead of pesticides.

In total, 79% of health workers claimed to have never followed a course or training module on pesticide poisoning management, even in the medical studies curriculum. Some had done courses on the management of food intoxications and snake bites.

For all the healthcare settings visited, 56 cases of pesticide poisoning were declared between 2011 and September 2016. In addition, 58% (*n* = 38) of health personnel interviewed claimed to be aware of other suspected poisoning cases in other health facilities with which they collaborated. In total, 78% of poisoning cases were accidental, 12% suicide attempts, 4% criminal (in the area of Njombe–Penja, thieves, when arrested, are injected with Gramoxone).

In more than 60% of cases, the pesticide products suspected to be the cause of the accidents could not be identified. The most frequently identified pesticide active ingredients in poisoning cases were cypermethrin, glyphosate, paraquat and metalaxyl. The most recurrent product names were Roundup (16%), Gramoxone (13%) and “Timides” (11%). “Timides” can also be considered as a non-identified product, as it is a common name used to designate insecticides in general.

With regard to the age of patients (*n* = 31), 19% were children, 26% adolescents and 55% adults. As for the gender, 73% of suspected patients of pesticide poisoning were male.

Circumstances of poisoning vary: 27% during spraying, 20% by ingestion (drinking, food contaminated by hands that have been used to manipulate pesticides), 13% occurred at home, 7% in the kitchen, and 3% during fishing.

Clinical signs said to be shown by patients included vomiting (18%), salivation (11%), indistinct vision (11%), respiratory difficulties (9%), convulsions (7%), asthenia (7%), dizziness (6%), headache (6%) and stomach problems (6%), among others.

In total, 71% of cases were hospitalized between 1 and 14 days, with 79% who recovered and 21% who died.

Treatment varied from case to case depending on the situation of the patients, category of the centers, the level of preparedness of the healthcare center and the knowledge of the health worker who handled the patient. A synopsis of recurrent treatments and drugs delivered to 32 patients in the health settings visited included (i) administration of antihistamine, antibiotic and anti-inflammatory; (ii) administration of atropine, stomach washing; (iii) drip, administration of dexamethasone and atropine; (iv) administration of bicarbonate and rehydration salts; (v) administration of corticoids and antibiotic drugs; (vi) dexamethasone, anti-inflammatory drugs and amoxicillin; (vii) dexamethasone and non-sugar milk; (viii) red oil administration; (ix) use of a ringer solution and cymethidin injection. Because of the absence of standardized management procedures, health personnel act according to their understanding of the case. We found that certain rural health centers collected empty packages of some pesticides used by farmers in their localities and used them to identify suspected products when a patient arrived.

## 4. Discussion

Pesticides are not manufactured locally; instead, they are imported from European and African countries. In this study, a total of 180 different pesticides were identified out of the 610 approved for use in Cameroon [3]. Among them, some unauthorized pesticides coming from neighboring countries were found: from Nigeria (Para Q, Multhrin 100, Maizine, Multiphos, PIAPIA, Shantisol liquid, EMSATE, Diastar, Pentagon, Orizo plus); others from Chad (Conquest), commonly known as Coton Tchad. Those products were essentially found in the northern part of the country, which has an important common border with Nigeria. In addition, expired pesticides and pesticides made locally were also found to be sold on the market [6].

For many pesticides, there is confusion between prohibited and newly approved ones due to similar product names (Bastion 10 G forbidden and Bastion Super approved; Cyplamdim 260 EC forbidden and Cyplandim Super approved; le Malagrain DP 5 forbidden and Malagrain Super approved; Furaplant 10 G forbidden and Furaplant Super approved).

In rural areas, pesticides are used on products for which they are not approved. This can be explained by the important demand of these products by farmers in rural settings [3,6,9].

Another practice observed is that, most of the time, farmers do not notice the differences in the name and composition of products; for instance, all paraquat pesticides are commonly called Gramoxone; because they have been used to that, and despite the fact that Gramoxone is no longer found on the market, they still ask for it and are given other paraquat pesticides. Therefore, we believe that in realizing a pesticide survey in Cameroon, care should be taken while interviewing farmers to avoid overestimating uses of certain products.

The abundance of pesticide products on local markets, coupled with farmers’ and salesmen’s pesticide risk perception, low level of education as well as inappropriate training, lead to product overuse and misuse. These situations have been pointed out as key pesticide-exposure risk factors [6,11,13,14]. In a study on farmers’ perception of agrochemicals in Nigeria, the levels of education, age and farming experience were also proven to be important determinants in pesticide use and exposure [15,16]. The present study indicates that inhalation and ingestion are perceived by more than 50% of farmers as the most important routes of pesticide exposure. We found that pesticide inhalation happened during spraying, while ingestion is mostly through the consumption of contaminated foods and water. This observation complies with conclusions from a community cross-sectional study conducted in Ethiopia in 2016. In that study, Gesesew et al. indicated that the probability of pesticide exposure was very high, and the two most important routes of exposure as reported by farmers were inhalation and ingestion [17,18]. The most perceived human health concerns were skin irritation, deaths, and respiratory and eye problems, which were also reported in other investigations [6,9,10,11,12,13,14,15,16,17,18,19]. Although people interviewed in our study did not name asthma as a pesticide health problem, 24% of farmers in the study in Ethiopia [17] cited asthma as a serious health problem. In an opinion article, some evidence has been gathered to suggest the serious risk of asthma among farmers exposed to high levels of pesticides [20]. Acute pesticide poisoning of farmers and relatives occurs mostly via inhalation [2], while risks of long-term effects may occur through ingestion of pesticide residues in foods [7,8,9,10,11,12,13,14,15,16,17,18,19,20,21]. Once in the human body, pesticides can, via different biochemical pathways, lead to chronic diseases such as cancer, diabetes, birth defects, chronic obstructive pulmonary disease and reproductive disorders [22]. More attention should also be paid to emerging pesticides causing transgenerational health effects on unborn children. Some pesticides are known to be endocrine-disrupting compounds, and as such, can alter the normal functioning of the endocrine system of both wildlife and humans [23]. Atrazine, for instance, is an endocrine-disrupting compound; its effects on the hypothalamic–pituitary–gonadal (HPG) axis have been examined [24]. Once poisoning has occurred, cases are often managed by using local unproven practices. Washing with clean water and soap is an accepted practice, while other studies revealed similar practices, including drinking milk and using red palm oil to accelerate wound healing [6,9,10,11,12,13,14]. Some poisoning cases, however, arrived at the nearest health center where often, the level of preparedness to face pesticide poisoning is very poor [4]. Current national health policy assumes that pesticide diseases are not a serious concern, partly because few data exist to provide a proper snapshot of the local and national burden of agricultural pesticides. Improvements in surveillance systems should involve the restructuring of data collection and facilitation of stakeholder collaboration. Cameroon could follow the example of some countries with pesticide surveillance systems [4,5,6,7,8,9,10,11].

## 5. Conclusions

Data from this study demonstrate that pesticides are widely used for exported and locally sold agricultural products. Different types of pesticides and formulations are distributed in all agro-ecological zones of Cameroon. Agrochemicals used by farmers are mostly imported, and their use does not always adhere to recommended safe practices. Pesticide distributors are major intermediates who make products available in remote areas of the country. Farmers who are the end users of pesticides complain of often being exposed, and cannot find appropriate cures. Health centers’ and workers’ levels of preparedness remain very weak and require significant improvement.

## Figures and Tables

**Table 1:** Enumeration of the main distribution sites and neighboring villages surveyed.

Region	Period (2016)	Department	Main Sites	Surrounding Villages	Main Agricultural Products
Centre	27–29 July	Lékié, Mfoundi Nyong et Foumou	Obala, Awae, Yaounde	Obala, Mbele, Mbei, Mboa I, Minkama, Nkolbene	Cocoa
Ouest	4–6 August	Noun	Foumbot	Foumbot, Kouptamo Fosset, Koudouben, Mangoun	Tomatoes, maize
Littoral	9–11 August	Moungo	Njombe-Penja	Njombe, Penja	Bananas
Nord-Ouest	18–20 August	Mezam	Santa	Njong, Santa, Ndikum, Pinying	Potatoes, celery, basil, leek, tomatoes
Nord	4–10 September	Benoue	Ngong	Tcheboa, Djefaltou, Ngong, Massa, Malla, Ndjole-Kapsiki	Peanuts, cotton, maize, pea, yam

**Table 2:** Number of persons surveyed in the three tiers (salesmen, farmers, health personnel).

Distribution of Persons Surveyed			Total
Salesmen	Health Personnel	Farmers	
18	10	84	112
21	12	103	136
14	5	75	94
6	9	64	79
10	4	86	100
69	38	412	

**Table 3:** Age of farmers across the five agro-ecological zones.

Survey Site	Ngong–Graoua	Njombe–Penja	Foumbot	Santa	Obala
Mean age	40 ± 11	44 ± 11	44 ± 10	49 ± 14	52 ± 12
Min.	22	20	21	23	21
Max.	63	71	79	75	75

**Table 4:** The five most produced items for each surveyed site.

Zones	Main Produce (Top 5) in %					Cumulative %
	1	2	3	4	5	
Obala	Cocoa (30.9%)	Maize (10.1%)	Plantain (9.7%)	Peanut (9.2%)	Banana (8.3%)	68.2
Foumbot	Tomato (20.3%)	Maize (16.0%)	Bean (15.2%)	Chilli pepper (12.1%)	Vegetable (10.2%)	73.8
Santa	Potato (21.7%)	Bean (17.7%)	Maize (16.6%)	Cabbage (12.6%)	Carrot (11.4%)	80
Njombe–Penja	Cocoa (25.7%)	Plantain (17.5%)	Papaya (12%)	Pineapple (9.3%)	Macabo (8.7%)	73.2
Ngong–Garoua	Peanut (31%)	Maize (27.8%)	Cotton (8.5%)	Rice (7.3%)	Cowpea (6.9%)	81.5

**Table 5:** Most used pesticides.

Zones	Most Used Pesticides (Top 5) in %					Cumulative %
	1	2	3	4	5	
Obala	Ridomil (8%)	Onex (6.3%)	Actara (5.7%)	Callomil (5%)	Lamida Gold, Parastar and Plantomil (4.7% each)	39.4
Foumbot	Gramoxone (12.6%)	Timide (7.4%)	K-optimal (7.1%)	Roundup (6.7%)	Cigogne and Pencozeb (4.1% each)	42
Santa	Gramoxone (12.7%)	Banko (12%)	Pencozeb (8%)	Roundup and Mancozan (6.7% each)	Parastar (4.7%)	50.8
Njombe–Penja	Gramoxone (12.8%)	Pyriforce (9.4%)	Glyphader (7.2%)	Ridomil and Supraxone (4.7% each)	Roundup and Capsidor (4.3% each)	47.4
Ngong–Garoua	Roundup (23.7%)	Atrazine (20.1%)	Gramoxone (15.9%)	Diuron (14.8%)	Biosec Roundup 720 (3.2%)	80.9

**Table 6:** Distribution of poisoning cases reported.

Zone	Case of Accidents Experienced by Farmers				Total
	Statistic	Cutaneous Contact	Ingestion	Inhalation	
Obala	Number	35	1	6	42
	%	83.3	2.4	14.3	100.0
Njombe–Penja	Number	31	0	4	35
	%	88.6	0.0	11.4	100.0
Ngong–Garoua	Number	21	0	4	25
	%	84.0	0.0	16.0	100.0
Santa	Number	9	0	2	9
	%	81.8	0.0	18.2	100.0
Foumbot	Number	37	0	6	37
	%	86.0	0.0	14.0	100.0
Total		133	1	22	156

**Table 7:** Most incriminated pesticides by farmers.

Zones	Most Incriminated Pesticides by Farmers (Top 5) and Exposition Routes					Number of Cases
	1	2	3	4	5	
Obala and Awae	11 cases with products not identified (8 cutaneous, 1 inhalation and 2 ingestion)	**Gramoxone**, 5 cases (3 inhalation and 2 cutaneous)	**Lamida gold**, 5 cases (3 cutaneous, 2 inhalation	**Dinacacao 40 EC (emulsifiable concentrate)**, 2 cases (1 inhalation, 1 cutaneous); **Beauchamp**, 2 cases (1 ingestion, 1 eye contact)	**Actara**, 2 cases (cutaneous)	46 (27 + 19 other products with 1 case each)
Foumbot	**Gramoxone**, 10 cases (8 cutaneous, 1 inhalation, 1 ingestion)	8 cases with products not identified (6 cutaneous, 1 inhalation and 1 ingestion)	**“Timide”,** 4 cases (3 cutaneous and 1 inhalation)	**Cygogne 360**, 3 cases (2 cutaneous and 1 eye contact) and a mixture **Cygogne + Cypercote**, 3 cases (2 cutaneous and 1 inhalation)	**K-optimal**, 2 cases (2 cutaneous)	40 (30 + 10 other products with 1 case each)
Santa	6 cases with products not identified	**Gramoxone**, 3 cases (cutaneous)	**Roundup**, 2 cases (inhalation)	**Mancozan**, 2 cases (1 cutaneous, 1 eye contact)	**Parastar** and **Dimeforce**, 1 case each (eye contact)	15
Njombe–Penja	10 cases with products not identified (6 cutaneous, 2 inhalation, 1 ingestion and 1 eye contact)	**Gramoxone**, 7 cases (4 cutaneous, 1 inhalation, 2 eye contact)	**Cigogne 360**, 4 cases (2 cutaneous, 2 inhalation)	**Metalm**, 2 cases (1 cutaneous and 1 inhalation)	10 others, different pesticides with 1 case each	33
Ngong–Garoua	7 cases with products not identified (5 cutaneous, 2 inhalation)	**Roundup**, 6 cases (5 cutaneous and 1 inhalation)	**Gramoxone** 4 cases (2 inhalation, 1 cutaneous and 1 eye contact)	**Thioral**, 4 cases (cutaneous)	**Atrazine**, 3 cases (2 cutaneous and 1 inhalation)	24

## References

[1] FAO and WHO (2014): The International Code of Conduct on Pesticide Management. http://www.fao.org/fileadmin/templates/agphome/documents/Pests_Pesticides/Code/CODE_2014Sep_ENG.pdf.

[2] Nirmala B., Langley R., Buhler W., Brantham K. (2016). Contributing factors for acute illness/injury from childhood pesticides exposure in North Carolina, USA, 2007–2013. Toxics.

[3] Ministry of Agriculture and Rural Development, National Registration Commission of Phytosanitary Products and Certification of Sprayers (2015) Liste des Pesticides Homologues au Cameroun au 27 Novembre 2015. http://www.reca-niger.org/spip.php?article579.

[4] Bertrand P.G., Ahmed H.A.M., Ngwafor R., Frazzoli C. (2016). Toxicovigilance Systems and Practices in Africa. Toxics.

[5] Gimou M.-M., Charrondiere U.R., Leblanc J.C., Pouillot R. (2008). Dietary exposure to pesticide residues in Yaoundé: The Cameroonian total diet study. Food Addit. Contam..

[6] Matthews G., Wiles T., Baleguel P. (2003). A survey of pesticide application in Cameroon. Crop Prot..

[7] Sonchieu J., Benoit Ngassoum M., Bosco Tchatchueng J., Srivastava A.K., Srivastava L.P. (2010). Survey of pesticide residues in maize, cowpea and millet from northern Cameroon: Part I. Food Addit. Contam. Part B.

[8] Kamga A., Kouame C., Tchindjang M., Chagomoka T., Drescher A.W. (2013). Environmental impacts from overuse of chemiclas fertilizers and pesticides amongst market gardening in Bamenda, Cameroon. Rev. Sci. Tech. For. Environ. Bassin Congo.

[9] Tandi T.E., Wook C.J., Shendeh T.T., Eko E.A., Afoh C.O. (2014). Small-Scale Tomato Cultivators’ Perception on Pesticides Usage and Practices in Buea Cameroon. Health.

[10] Tarla D.N., Manu I.N., Tamedjouand Z.T., Kamga A., Fontem D.A. (2015). Pligth of pesticide applicators in Cameroon: Case of Tomato (*Lycopersiconesculentum Mill*) farmers in Foumbot. J. Agric. Environ. Sci..

[11] London L., Bailie R. (2001). Challenges for improving surveillance for pesticides poisoning: Policy implications for developing countries. Int. J. Epidemiol..

[12] Konradesen F., van der Hoek W., Cloe D.C., Hutchinson G., Daisley H., Singh S., Eddleson M. (2003). Reducing acute poisoning in developing countries: Options for restricting the availability of pesticides. Toxicology.

[13] McCauley L.A., Kent Anger W., Keifer M., Langley R., Robson M.G., Rohlman D. (2006). Studying health outcomes in farmworker population exposed to pesticides. Environ. Health Perspect..

[14] Tarla D.N., Meutchieye F., Assako V.A., Fontem D.A., Kome J.J.A. (2013). Exposure of market gardeners during pesticide application in the western highlands of Cameroon. Sch. J. Agric. Sci..

[15] Issa F.O. (2016). Farmers Perception of the Quality and Accessibility of Agrochemicals in Kaduna and Ondo States of Nigeria: Implications for Policy. J. Agric. Ext..

[16] Okolle N.J., Afari-Sefa V., Bidogeza J.-C., Tata P.I., Ngome F.A. (2016). An evaluation of smallholder farmers’ knowledge, perceptions, choices and gender perspectives in vegetable pests and diseases control practices in the humid tropics of Cameroon. Int. J. Pest Manag..

[17] Gesesew H.A., Woldemichael K., Massa D., Mwanri L. (2016). Farmers Knowledge, Attitudes, Practices and Health Problems Associated with Pesticide Use in Rural Irrigation Villages, Southwest Ethiopia. PLoS ONE.

[18] Damalas C.A., Koutroubas S.D. (2016). Farmers’ Exposure to Pesticides: Toxicity Types and Ways of Prevention. Toxics.

[19] Tagwireyi D., Chingombe P., Khoza S., Maredza M. (2016). Pattern and Epidemiology of Poisoning in the East African Region: A Literature Review. J. Toxicol..

[20] Amaral A.F.S. (2014). Pesticides and asthma: Challenges for epidemiology. Front. Public Health..

[21] Jean S., Benoit N.M., Bosco T.J., Srivastava A.K., Srivastava L.P. (2013). Contamination of cowpea and by-products by organophosphorus pesticide residues in Ngaoundere markets: Dietary risk estimation and degradation study. Afr. J. Food Sci..

[22] Mostafalou S., Abdollahi M. (2013). Pesticides and human chronic diseases: Evidences, mechanisms, and perspectives. Toxicol. Appl. Pharmacol..

[23] Mnif W., Hassine A.I.H., Bouaziz A., Bartegi A., Thomas O., Roig B. (2011). Effect of Endocrine Disruptor Pesticides: A Review. Int. J. Environ. Res. Public Health.

[24] Sara E.W., Jennifer L.F. (2015). Atrazine Exposure and Reproductive Dysfunction through the Hypothalamus-Pituitary-Gonadal (HPG) Axis. Toxics.

